# *Lasiodiplodia* sp. ME4-2, an endophytic fungus from the floral parts of *Viscum coloratum*, produces indole-3-carboxylic acid and other aromatic metabolites

**DOI:** 10.1186/s12866-014-0297-0

**Published:** 2014-11-30

**Authors:** Chao-Dong Qian, Yu-Hang Fu, Fu-Sheng Jiang, Zheng-Hong Xu, Dong-Qing Cheng, Bin Ding, Cheng-Xian Gao, Zhi-Shan Ding

**Affiliations:** Zhejiang Chinese Medical University, Hangzhou, Zhejiang province China

**Keywords:** *Lasiodiplodia* sp, Floral endophytes, Mistletoe, Aromatic compounds

## Abstract

**Background:**

Studies on endophytes, a relatively under-explored group of microorganisms, are currently popular amongst biologists and natural product researchers. A fungal strain (ME4-2) was isolated from flower samples of mistletoe (*Viscum coloratum*) during a screening program for endophytes. As limited information on floral endophytes is available, the aim of the present study is to characterise fungal endophytes using their secondary metabolites.

**Results:**

ME4-2 grew well in both natural and basic synthetic media but produced no conidia. Sequence analysis of its internal transcribed spacer rDNA demonstrated that ME4-2 forms a distinct branch within the genus *Lasiodiplodia* and is closely related to *L. pseudotheobromae*. This floral endophyte was thus identified as *Lasiodiplodia* sp. based on its molecular biological characteristics. Five aromatic compounds, including cyclo-(Trp-Ala), indole-3-carboxylic acid (ICA), indole-3-carbaldehyde, mellein and 2-phenylethanol, were found in the culture. The structures of these compounds were determined using spectroscopic methods combined with gas chromatography. To the best of our knowledge, our work is the first to report isolation of these aromatic metabolites from a floral endophyte. Interestingly, ICA, a major secondary metabolite produced by ME4-2, seemed to be biosynthesized via an unusual pathway. Furthermore, our results indicate that the fungus ME4-2 is a potent producer of 2-phenylethanol, which is a common component of floral essential oils.

**Conclusions:**

This study introduces a fungal strain producing several important aromatic metabolites with pharmaceutical or food applications and suggests that endophytic fungi isolated from plant flowers are promising natural sources of aromatic compounds.

## Background

Endophytes refer to microorganisms that asymptomatically colonise the internal tissues of plants for at least a part of their life cycle. Each plant on earth, from the arctic tundra to the tropics, has been estimated to contain at least one endophyte [1,2]. Fungi and bacteria are the most common microbes existing as endophytes and presumed to have originated from the external environment, although some endophytes are vertically transmitted to succeeding plant generations via seeds [1,3]. A number of these microorganisms have intimate interactions with their host plants and can promote plant growth as well as enhance host adaptation to biotic and abiotic stress factors [2,4,5]. In some cases, the beneficial effects of endophytes are exhibited through production of bioactive secondary metabolites, such as plant hormones (e.g., auxins and cytokinins), adenine ribosides and defence-related compounds [6–8].

Although endophytes were discovered as early as 1904, they did not receive significant attention until the isolation of taxol from an endophytic fungus *Taxomyces andreanae* in 1993 [9]. Endophytes have since then been shown to produce several other well-known and important plant-derived compounds, such as camptothecin [10], isocoumarin [11] and podophyllotoxin [12], amongst others [13–15]. Although Heinig *et al*. [16] re-examined and found no evidence of independent taxane biosynthesis in any of the endophytes associated with *Taxus* species in 2013, it is believed that endophytes will become alternative sources for pharmacologically important natural products from plants [15,17]. In addition, endophytes can also produce a wide range of novel natural compounds with various biological activities [13–15,18]. As a significantly untapped reservoir of functional metabolites, endophytes are fairly under-utilised [19]. As such, additional investigations are necessary to discover new endophytes with potential applications [15,20,21].

**Figure 1:**
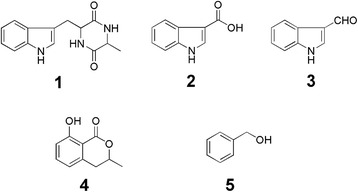
**Compounds isolated from fermentation cultures of** ***Lasiodiplodia*** **sp. ME4**-**2. 1**, cyclo-(Trp-Ala); **2**, indole-3-carboxylic acid; **3**, indole-3-carbaldehyde; **4**, mellein; **5**, 2-phenylethanol.

Mistletoe *Viscum coloratum*, a semi-parasitic evergreen dioecious shrub, is an important medicinal herb used in Traditional Chinese Medicine mainly for treating rheumatic arthralgia and foetal upset [22]. Although mistletoe and its active secondary metabolites have been widely studied [23–25], research on endophytes from this herb is virtually unavailable. Endophytic fungal communities in the semi-parasitic epiphyte *V. album* and its phorophyte *Pinus sylvestris* were recently compared and studied [26,27]; however, no *Viscum* endophyte was isolated and screened for active compounds. During a screening program for endophytic microbes hosted by *V. coloratum*, an endophytic fungus, ME4-2, was isolated from the floral parts of the plant. The aim of the present study is to characterise the floral endophyte together with its active compounds. The major metabolites produced by the strain are aromatic compounds, including cyclo-(Trp-Ala) (1), indole-3-carboxylic acid (ICA) (2), indole-3-carbaldehyde (3), mellein (4) and 2-phenylethanol (5) (Figure 1), all of which are valuable compounds for the pharmaceutical and food industries, as well as host close related metabolites.

## Results and discussion

### Identification of endophytic fungus ME4-2

Six segments of each floral sample were placed on potato dextrose agar (PDA) and screened for the presence of endophytic fungi. ME4-2 was consistently isolated from all samples and selected for further studies. ME4-2 grew well on PDA and Czapek’s media. Cultures were initially white with moderately dense aerial mycelium and then turned grey after 4 d with thick aerial mycelium. The mycelium consisted of smooth, branched, septate and subhyaline hyphae. No conidium was observed under the test conditions employed, including cultivation on water agar (WA) with pine needles or mistletoe twigs for 45 d or at room temperature with diffused daylight. The optimum temperature for strain growth was 30°C, and the strain reached 90 mm in diameter on PDA and synthetic low nutrient agar (SNA) after 48 and 96 h, respectively.

**Figure 2:**
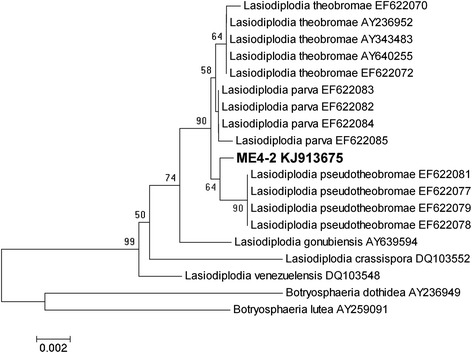
**Unrooted neighbour**-**joining tree for ME4**-**2 within** ***Lasiodiplodia*** **species.** Numbers at nodes indicate bootstrap values (expressed as percentages of 1000 replications); Values lower than 50% are not indicated. Scale bar = 0.002 substitutions per nucleotide position.

To acquire phylogenetic information on the fungus, the DNA sequence of the internal transcribed spacer (ITS) regions of ME4-2 was amplified and sequenced. The PCR products included approximately 520 base pairs. Unreliable sequence data from the ends of the sequences were excluded and 484 characters (GenBank Accession No. KJ913675) were used during analysis. A phylogenetic tree was constructed (Figure 2) based on the ITS sequences of ME4-2 and closely related strains. ME4-2 formed a distinct branch within the genus *Lasiodiplodia* and was closely related to *L. pseudotheobromae*. Molecular analysis indicated that ME4-2 is a new member of the genus *Lasiodiplodia*. Previous research [28] showed that *Lasiodiplodia* sp. is one of the more common endophytes found in marketed flowers, consistent with our findings. We note, however, that the use of a single ITS sequence is insufficient to achieve complete analysis. To establish the taxonomic status of the strain with high precision, three or more genes are necessary to construct phylogenetic trees.

### Secondary metabolites produced by strain ME4-2 in broth culture

To characterise *Lasiodiplodia* sp. ME4-2 further, secondary metabolites produced by the strain cultured in Czapek’s broth for 6 d were isolated and identified. Four aromatic compounds with relatively high concentrations were obtained and identified as cyclo-(Trp-Ala) (1), ICA (2), indole-3-carbaldehyde (3) and mellein (4). An additional trace but detectable compound, namely, 2-phenylethanol (5), was also found using gas chromatograph-mass spectrometry (GC-MS).

Compound 1 was determined as cyclo-(Trp-Ala) (mass spectrum: m/z 256, [M-H]^−1^) by nuclear magnetic resonance (NMR) analysis and comparison of its date with those reported in the literature [29]. The compound is known to be isolated from many microorganisms and shows weak antioxidant activity [30].

Compounds 2 and 3 were identified as ICA and indole-3-carbaldehyde, respectively, through comparison with authentic samples (Table 1). The molecular structures of the compounds were further confirmed through their NMR spectra. Both compounds are commonly present in microorganisms and plants and present antimicrobial and antitumor activities [31,32]. The biological role of ICA, which is regarded as an inactive auxin (indole-3-acetic acid, IAA) analogue, in plants has long been neglected. Recent studies indicate that ICA may perform important functions in plant basal defence against biotic stress, and has been identified as a mediator of induced resistance in *Arabidopsis* against plant pathogens [33,34]. Endophytes are known to produce bioactive compounds that protect their plant hosts from various pathogens. However, whether or not ICA is actually produced in the plant and whether or not the compound produced by ME4-2 is beneficial to its host have yet to be determined.

**Table 1:** **GC**/**MS analysis of the compounds produced by ME4**-**2 after 6 days of incubation at 28**°C **in Czapek**’**s broth**

**R**_**t**_ **(min)**	**molecular mass (Da)**	**Possible compounds**	**Standards** ^**a**^	**Similarity (%)** ^**b**^
8.95	122	2-phenylethanol	a	98
13.25	117	Indole	a	95
17.67	178	Mellein		89
22.91	145	Indole-3-carbaldehyde	a	94
24.80	161	Indole-3-carboxylic acid	a	94
34.94	204	Tryptophan	a	90
35.29	257	Cyclo-(Trp-Ala)		90

^a^Denotes that the retention time and MS spectrum were identical to or closely matched those of an authentic standard compound.

^b^Denotes the similarity between the mass spectrum of a secondary metabolite produced by ME4-2 and the most closely matched compound in the National Institute for Standards and Technology (NIST) database.

Compound 4 was identified as mellein based on GC-MS and NMR data. The GC-MS spectrum of 4 showed a molecular ion peak at m/z 178, which is identical to that of mellein. The UV, ^1^H NMR and ^13^C NMR data of 4 were also consistent with those reported for the known compound [35,36]. Whilst mellein has been previously isolated from various fungi, including plant pathogens and endophytes, the present study is the first to report mellein isolation from a floral endophytic fungus. Mellein, originally identified as a phytotoxic compound, is a versatile compound with various biological activities, such as antibacterial, fungicidal, anti-worm and HCV protease-inhibitory [37]. Interestingly, the compound has been also isolated from various insect secretions as a trail or alarm pheromone [38].

**Figure 3:**
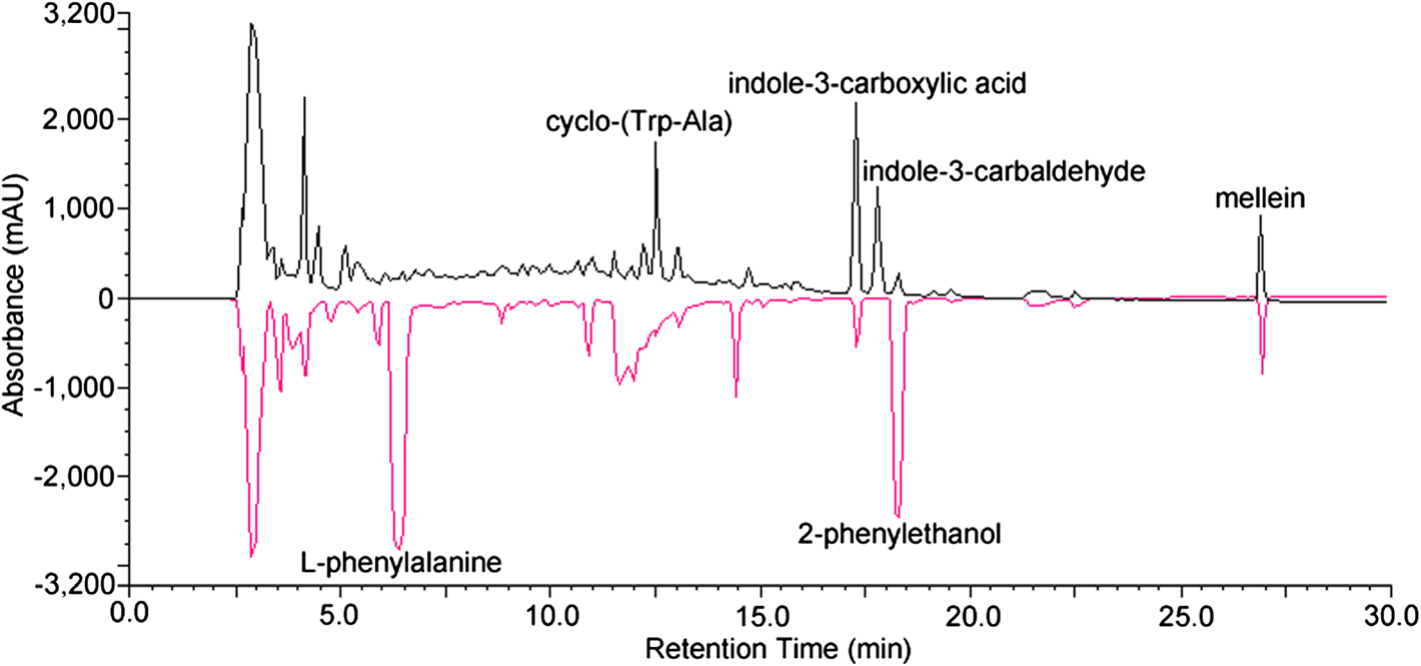
**Effect of L**-**phenylalanine supplementation to Czapek**’**s medium on 2**-**phenylethanol production.** The red and black curve represent for the fermentation supernatant with or without L-phenylalanine (6 g/L), respectively. UV absorption was measured at 210 nm.

Compound 5 was determined to be 2-phenylethanol through comparison with a commercially available standard (Table 1). The fragmentation pattern of the compound in its GC-MS spectrum as well as its behaviour during high-pressure liquid chromatography (HPLC) were identical to those of synthetic 2-phenylethanol. Compound 5 was a minor secondary metabolite produced by *Lasiodiplodia* sp. ME4-2 cultured in PDA and Czapek’s broth. Supplementing the basic growth medium with precursors can improve the yields of the microbial products [39]. To increase 2-phenylethanol production, ME4-2 was cultured in Czapek’s broth supplemented with 6 g/L L-phenylalanine, a well-known precursor of 2-phenylethanol. Addition of L-phenylalanine to Czapek’s medium significantly increased 2-phenylethanol contents (Figure 3), which reached 226 mg/L. By contrast, a low 2-phenylethanol content of around 7–35 mg/L was obtained when the medium contained yeast extracts, peptone, tryptophan or (NH_4_)_2_SO4 as an additional nitrogen source.

2-Phenylethanol, a well-known flavour and fragrance substance with a rose-like odour, naturally occurs in the essential oils of certain flowers, such as rose, narcissi, lilies and jasmine [40]. Natural 2-phenylethanol has also been isolated from several types of fungi, including yeasts, mushrooms and filamentous fungi [41]. It is now generally accepted that some endophytes can produce similar chemical compounds to those produced by their hosts [15]. However, few floral endophytes have been screened for essential oil components, such as 2-phenylethanol. Consumer preference for natural compounds has recently stimulated the search for microorganisms that can produce 2-phenylethanol. Isolation of *Lasiodiplodia* sp. ME4-2, a potential phenylethanol producer, indicates that flower endophytes may be important resources of natural flavours.

### ICA biosynthetic pathway in *Lasiodiplodia* sp. ME4-2

ICA is an important intermediate product for pharmaceutical and agricultural industries with an aromatic indole ring [42]. Interestingly, ME4-2 can synthesise ICA without tryptophan, which is believed to be a precursor of ICA. It has been suggested that tryptophan was converted to ICA via IAA in microorganisms [43]. Several holoparasitic dicotyledonous plants, however, can directly metabolise L-tryptophan to indole-3-carboxaldehyde, which is further converted to ICA [44]. The ICA biosynthetic pathway in ME4-2 was examined by analysing metabolic intermediates. The GC-MS spectrum of the fungal culture extract showed (Table 1) the presence of a peak at a retention time of 24.80 min, which corresponds well with standard ICA. Peaks with retention times of 13.25, 22.91 and 34.94 min corresponded to the retention times of authentic indole, indole-3-carbaldehyde and tryptophan, respectively. No IAA was detected in the culture medium. Whilst these results suggest that de novo ICA biosynthesis in ME4-2 may occur via an IAA-independent route, more studies are necessary to confirm this hypothesis.

## Conclusion

In this study, a fungal endophyte *Lasiodiplodia* sp. ME4-2 from mistletoe flowers was isolated and identified using molecular characterisation results. *Lasiodiplodia* sp. ME4-2 is a newly isolated endophyte that can produce several interesting aromatic compounds, including cyclo-(Trp-Ala), ICA, indole-3-carbaldehyde, mellein and 2-phenylethanol, which are valuable compounds for the pharmaceutical and food industries. A pooled analysis of published literature together with our results suggests a close relationship between the strain and its floral host. ICA, a plant cell wall-bound metabolite that could mediate accelerated callose accumulation in response to pathogens, may enhance the host adaptation to various biotic stress factors. Mellein, whether as a phytotoxic compound or an alarm pheromone, may also affect the growth of its host plant. 2-Phenylethanol is a common component of flower essential oils. Although only small amounts of 2-phenylethanol are produced by ME4-2 cultured in synthetic media, production of this compound can be significantly increased by addition of L-phenylalanine to the culture broth. Interestingly, the major route of ICA biosynthesis in *Lasiodiplodia* sp. ME4-2 does not occur through IAA, which has been considered a precursor of ICA except in several rare cases. The present study introduces a fungal producer of several important aromatic compounds and enriches our knowledge of endophytes.

## Methods

### Fungal isolation and storage

Healthy flowers of the medicinal plant *Viscum coloratum* were collected from Hangzhou City, Zhejiang Province, China. Samples were placed in sterilised bags and kept on ice during transportation. All flowers were processed within 24 h of collection. The flowers were thoroughly washed in tap water and cut into small segments (about 1 cm × 1 cm). These segments were sterilised with 75% ethanol for 30 s and with 0.1% mercury dichloride for 1 min and then rinsed six times with sterile distilled water. Sterilised segments were placed on PDA and cultured at 28°C for 2–14 d to allow the growth of endophytic fungi. Individual colonies were then hyphal tipped and transferred to fresh PDA. Pure isolates were maintained on PDA at 28°C and stored at 5°C.

### Morphology characteristics

Morphological observations of the colonies were based on the isolated strain (ME4-2) grown on PDA and Czapek’s medium (3.0 g NaNO_3_, 1.0 g K_2_HPO_4_, 0.5 g MgSO_4_ · 7H_2_O, 0.5 g KCl, 0.01 g FeSO_4_ · 7H_2_O, 30.0 g sucrose, 20.0 g agar, and 1 L distilled water; pH 6.8) at 28°C. Microscopic observations of morphological features were performed using an Olympus BX51 microscope and DP71 digital camera. To induce sporulation, isolated ME4-2 was either grown on 2% WA with sterilised pine needles or mistletoe twigs placed in the medium incubated at 25°C in the dark or cultured at room temperature with diffused daylight [45]. Growth rates on PDA and SNA (1.0 g KH_2_PO_4_, 1.0 g KNO_3_, 0.5 g MgSO_4_ · 7H_2_O, 0.5 g KCl, 0.2 g glucose, 0.2 g sucrose, 1.0 L distilled water and 20.0 g agar) at 20, 25, 30, 35 and 40°C were determined using the method described by Chaverri *et al*. [46].

### DNA extraction, PCR amplification and sequencing

Isolated ME4-2 was grown in PDA for 4 d at 28°C. Genomic DNA was extracted from the mycelium according to the cetyltrimethylammonium bromide method [47]. The nuclear 5.8S ribosomal RNA gene and its flanking ITS regions were amplified using two universal primers, ITS1 and ITS4 [48]. The PCR products were separated by electrophoresis in 1% agarose gels and purified using a QIAquick gel exaction kit (Qiagen). The purified PCR products were then sequenced in both directions using the same primers used for PCR reactions. Sequence reactions were run on a Model 3730 automated capillary DNA sequencer (Applied Biosystems, Foster City, USA). The ITS sequence of strain ME4-2 was deposited in GenBank under the accession number KJ913675.

### Phylogenetic analysis

The ITS sequence of the endophytic fungus was analysed along with closely related sequences retrieved from GenBank using BLAST [49]. Sequences were aligned with Clustal W [50] and manually edited. A phylogenetic tree was constructed using the neighbour-joining method [51] with MEGA version 4.0 [52]. Tree topologies were evaluated using bootstrap analysis based on 1000 resamplings.

### Fermentation and compound isolation

ME4-2 was grown in 2 L Erlenmeyer flasks containing 800 mL of Czapek’s broth medium. Fermentation was conducted on a rotary shaker (120 rpm) at 28°C for 6 d. The culture broth (10 L) was filtered twice through six layers of conventional gauze, and the cell-free supernatant was loaded onto an AB-8 macroporous absorption resin column pre-equilibrated with distilled water. The column was washed with distilled water and eluted with 25% and 75% (v/v) methanol. The 75% methanol fraction was pooled and concentrated at 50°C using a rotary evaporator. The concentrated solution was centrifuged at 6000 rpm for 30 min, and the supernatant was transferred to a C_18_ SPE column (Hardwee, Germany). The column was washed with three bed volumes of distilled water followed by three bed volumes of a methanol/water mixture (20:80, v/v). The fraction eluted from the SPE column with a methanol/water mixture (60:40, v/v) was further concentrated and purified using semi-preparative HPLC on a Venusil XBP C_18_ (5 μm, 250 × 10 mm) column. HPLC was performed on a Dionex Ultimate 3000 HPLC System (Thermo Fisher Scientific, Waltham, USA) with a diode-array UV/VIS detector. The mobile phase was composed of an acetonitrile-water (0.05% formic acid) mixture and programmed using a linear gradient from 5% to 65% over 30 min. The UV detector was set to 210, 230, 260 and 280 nm, and the flow rate was 5 mL/min.

### Spectroscopic measurements

NMR spectra were recorded using an AVANCE DMX-600 spectrometer (Bruker, Karlsruhe, Germany) in dimethyl sulfoxide-d6 (1, 2 and 4) or acetone-d6 (3) at 27°C. Electrospray ionisation mass spectra were recorded in negative ion mode on a Thermo Finnigan LCQ mass spectrometer (Thermo Electron Corp., San Jose, USA).

### GC analyses

About 100 mL of culture broth (Czapek) was centrifuged at 10000 *g* for 20 min. The supernatant was extracted with 100 mL of chloroform, and the organic phase was concentrated to approximately 10 mL before GC analysis using a Focus GC-MS system (Thermo Scientific) equipped with an Rtx-5MS capillary column (30 m × 0.25 mm, 0.25 μm). The injector temperature was 250°C, and the flow rate of the helium carrier gas was 1.23 mL/min. The column temperature was maintained at 60°C for 3 min, raised to 180°C at 8°C/min, maintained at 180°C for 4 min, ramped to 300°C at 10°C/min and finally held for 5 min at 300°C. The data acquisition rate was 100 scans/s, and data were acquired over a mass range of 40–650 m/z. Compounds were tentatively identified by comparing their mass spectra with those available in the National Institute for Standards and Technology mass spectral library.

### Determination of the effect of nitrogen sources on 2-phenylethanol production

The growth medium (Czapek’s broth) was supplemented with different nitrogen sources to improve 2-phenylethanol production, and the effects of these sources on 2-phenylethanol production were studied. Culture supernatants were filtered through 0.2 μm filters prior to analysis. 2-Phenylethanol in the aqueous phase was analysed by HPLC on a Dionex C18 reversed-phase column (250 × 4.6 mm, 5 μm) over a linear gradient of 10% to 65% acetonitrile flowing at a rate of 1 mL/min for 30 min and UV detection at 210 nm.

## Abbreviations

ITS: Internal transcribed spacer
ICA: Indole-3-carboxylic acid
IAA: Indole-3-acetic acid
GC-MS: Gas chromatograph-mass spectrometer
PDA: Potato dextrose agar
WA: Water agar
NMR: Nuclear magnetic resonance
ESI-MS: Electrospray ionization-mass spectra
NIST: National institute for standards and technology
HPLC: High-pressure liquid chromatography
SNA: Synthetic low nutrient agar
